# Supplementary material for: Metagenomic surveillance uncovers diverse and novel viral taxa in febrile patients from Nigeria
Source: Nat Commun. 2023 Aug 4;14:4693. doi: 10.1038/s41467-023-40247-4 (PMC10403498; doi:10.1038/s41467-023-40247-4)
Supplement: Supplementary file 1 — Supplementary Information [file 41467_2023_40247_MOESM1_ESM.pdf]

Metagenomic surveillance uncovers diverse and novel viral taxa in febrile patients from Nigeria

Supplementary Note

We detected Lassa virus (LASV) via metagenomics in 3 samples that were negative for LASV via clinical RT-qPCR testing. To ensure that these samples were true false negatives, we thoroughly investigated their provenance.

- We re-tested the samples via RT-qPCR following metagenomic sequencing, and confirmed that they were RT-qPCR-negative (**Supplementary Figure 3**).
- We confirmed that the External RNA Controls Consortium (ERCC) RNA spike-ins were highly pure for these samples (>1.4 million reads assigned to ERCCs, of which >99.97% were assigned to the proper ERCC). This suggests that interwell contamination cannot explain these findings.
- We sequenced the samples using unique dual indexes, minimizing the likelihood of index hopping. They were sequenced on a different sequencing machine than the RT-qPCR-positive samples in our study, 4 months after any RT-qPCR-positive samples were processed in the laboratory.
- We compared the identical, complete LASV genomes that we produced from 2 of these samples to all LASV genomes present in NCBI GenBank and in our study. They were genetically distinct from all other available genomes.
- We analyzed the 3 genomes for mutations in the regions mapping to the Nikisins primers, which target the L gene[1]. The partial genome lacked coverage in the primer-binding regions. The 2 complete genomes possessed a mismatch at the second position of the forward primer (C**A**ACCATYTTRTGCATRTGCCA).
- We queried for epidemiological links between the 2 individuals with identical LASV genomes. Their samples were collected 2 days apart, though the individuals reside in different states and age brackets. However, we could not comprehensively rule out the possibility of human-to-human transmission.

Supplementary Figures

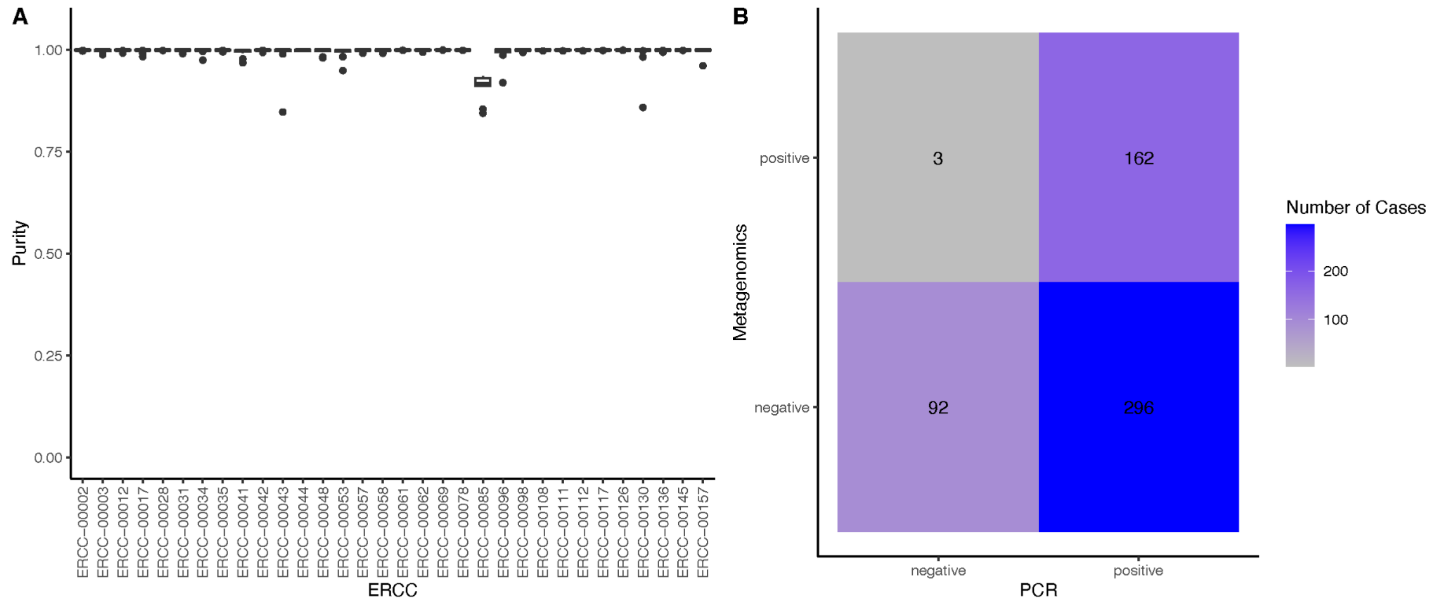

Supplementary Figure 1: Metagenomics requires stringent experimental processes and bioinformatic controls to accurately detect pathogens. A. Purity, i.e., percent of ERCC (External RNA Controls Consortium) reads assigned to the noted spike-in, vs. ERCC spike-in. N = 505 biologically independent samples, with 7-24 samples per ERCC. Boxplots display the first, second, and third quartiles, with whiskers extending to the data point that is maximally 1.5 times the interquartile range from the first (lower whisker) or third (upper whisker) quartile. **B.** Lassa virus metagenomic positivity vs. RT-qPCR positivity. $p < 0.001$ ($p = 1.61 \times 10^{-11}$) via two-sided Fisher's exact test.

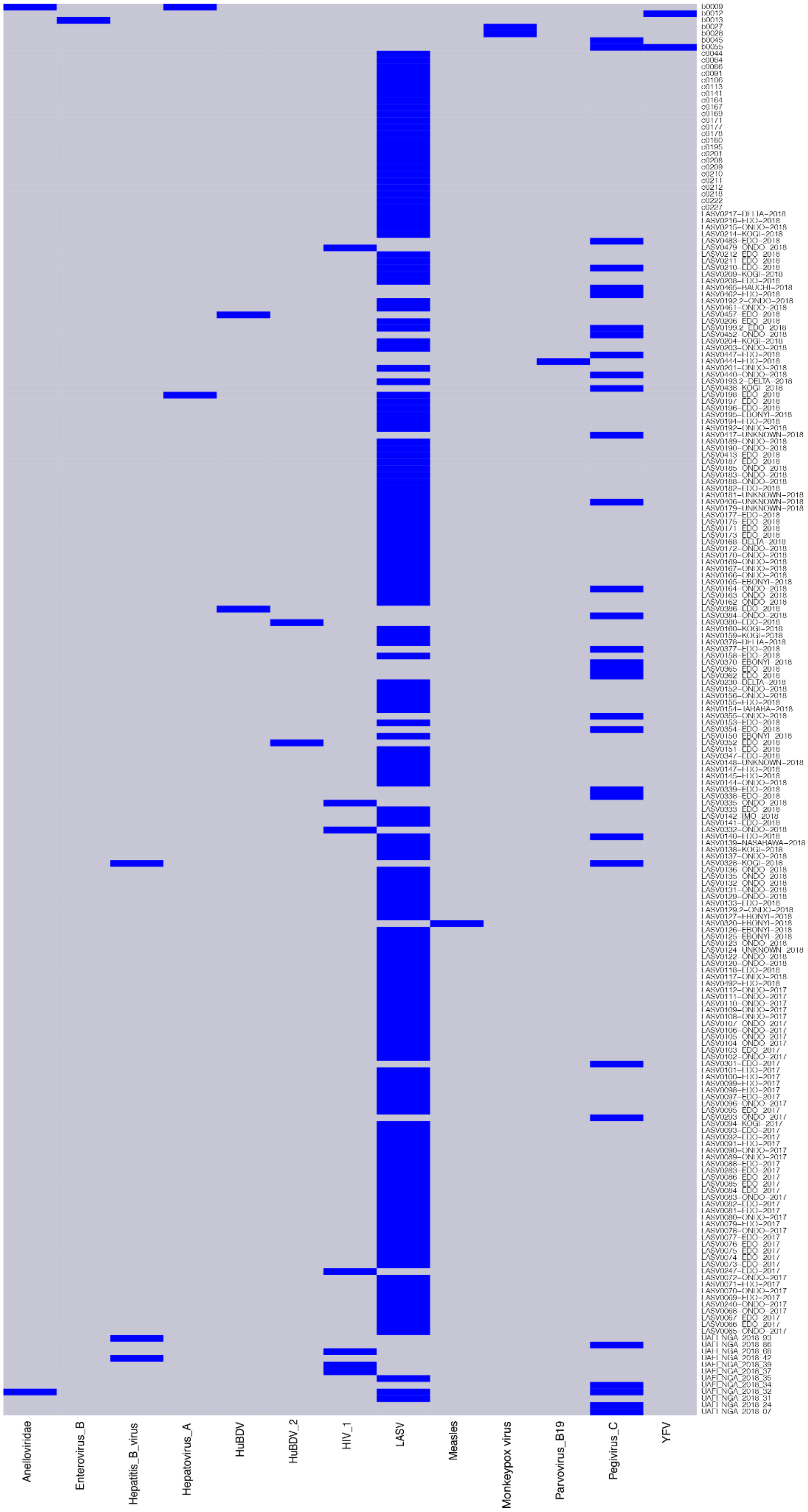

Supplementary Figure 2: Heat map of samples vs. viruses identified via metagenomic analyses.

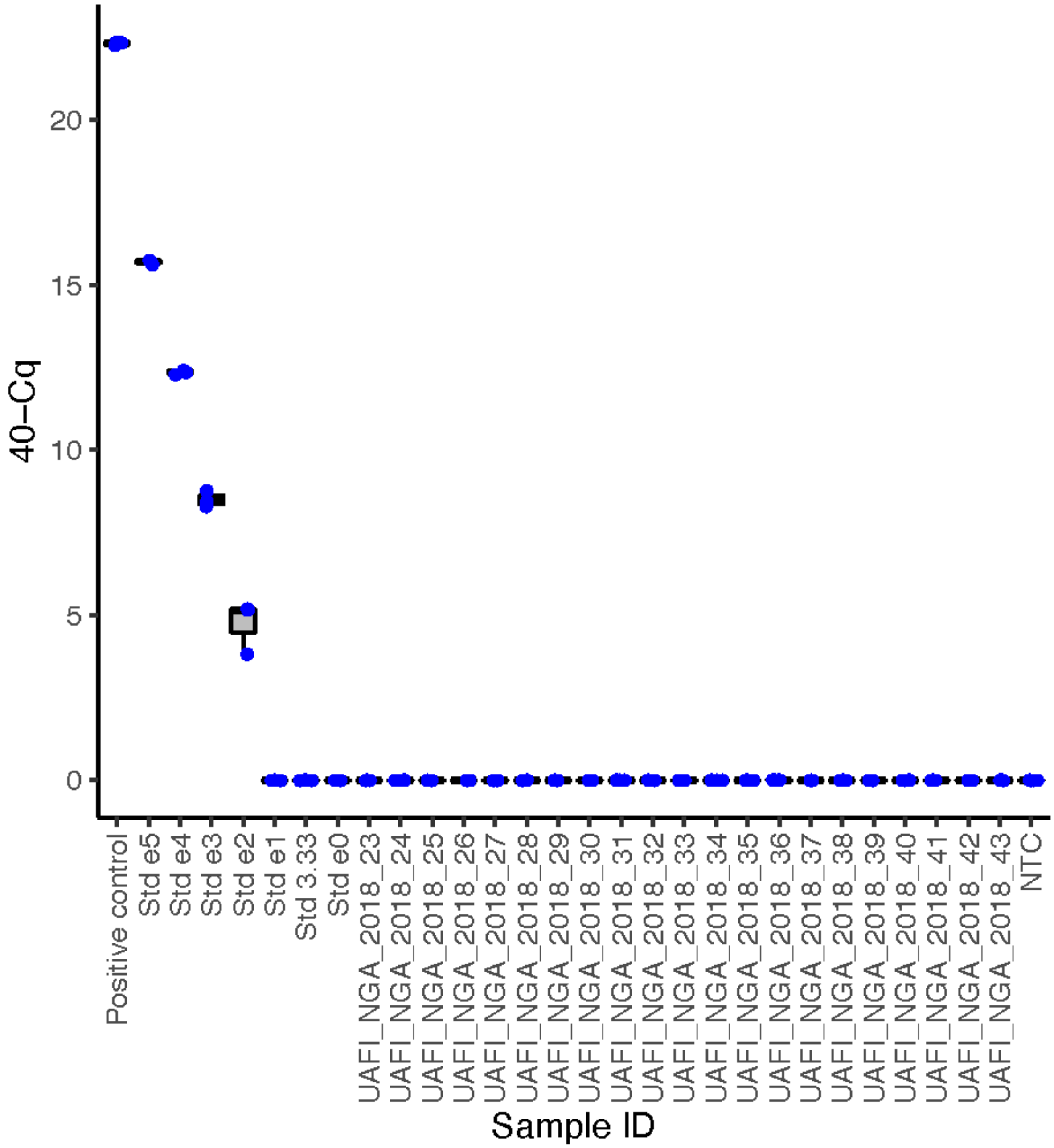

Supplementary Figure 3: RT-qPCR fails to identify Lassa virus (LASV) in 3 samples that yielded LASV genomes. Cycle threshold (Cq) values for LASV-negative samples, including the 3 samples for which partial or complete LASV genomes were produced. Samples were tested using the Nikisin primers. Std, standards. NTC, no-template control. N = 3 technical replicates per sample. Boxplots display the first, second, and third quartiles, with whiskers extending to the data point that is maximally 1.5 times the interquartile range from the first (lower whisker) or third (upper whisker) quartile.

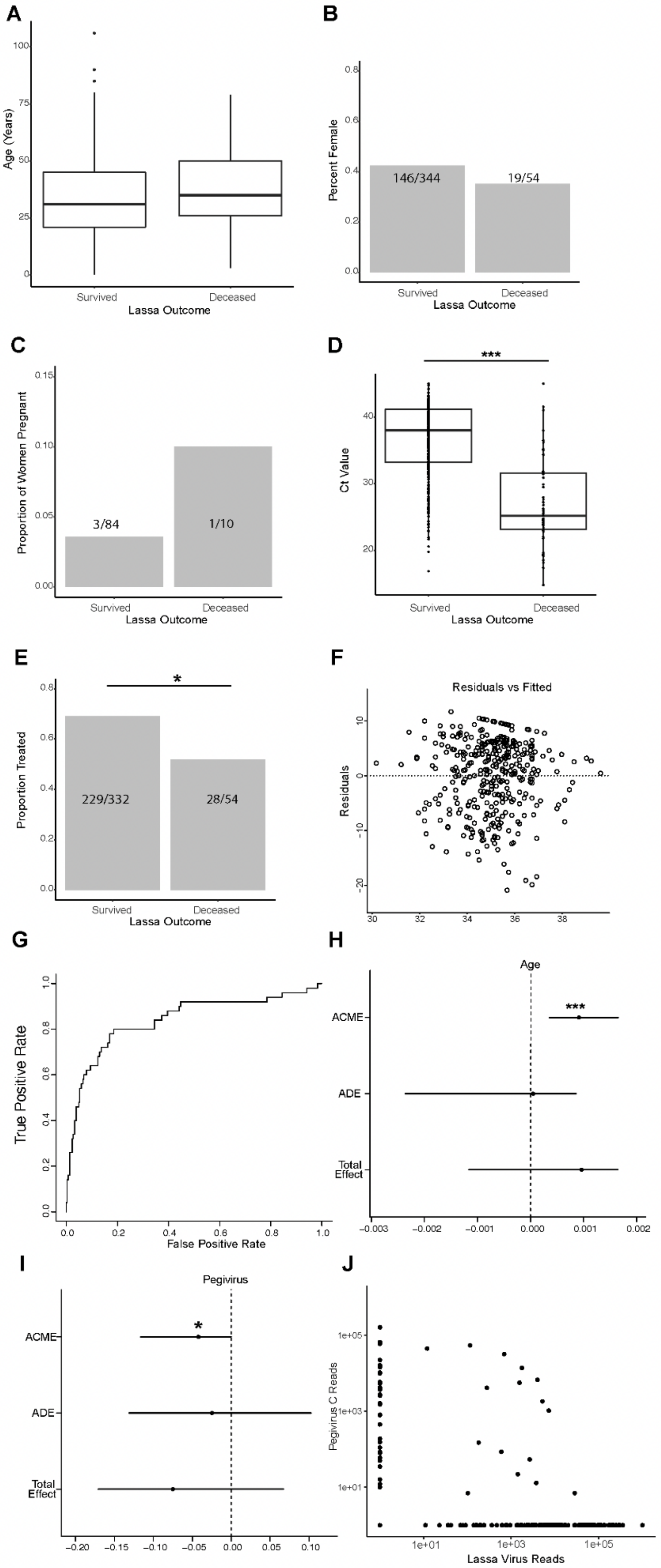
A
Age (Years)
Survived
Deceased
Lassa Outcome
B
Percent Female
146/344
19/54
Survived
Deceased
Lassa Outcome
C
Proportion of Women Pregnant
3/84
1/10
Survived
Deceased
Lassa Outcome
D
***
Ct Value
Survived
Deceased
Lassa Outcome
E
*
Proportion Treated
229/332
28/54
Survived
Deceased
Lassa Outcome
F
Residuals vs Fitted
Residuals
G
True Positive Rate
False Positive Rate
H
Age
***
ACME
ADE
Total Effect
I
Pegivirus
*
ACME
ADE
Total Effect
J
Pegivirus C Reads
Lassa Virus Reads

Supplementary Figure 4. Lassa Fever (LF) outcomes and causal mediation analysis. A-E. Distribution of predictor variables by LF outcome: age (**A**), sex (**B**), pregnancy (**C**; among females), cycle threshold value (**D**; Ct), and ribavirin treatment (**E**). N = 380 individuals (327 survived, 53 deceased; **A**). N = 391 individuals (340 survived, 51 deceased; **D**). P-values via univariate logistic regression (**D**, unadjusted $p = 2.79 \times 10^{-14}$; **E**, unadjusted $p = 0.01$). Boxplots display the first, second, and third quartiles, with whiskers extending to the data point that is maximally 1.5 times the interquartile range from the first (lower whisker) or third (upper whisker) quartile. **F.** Residual plot for the multivariate linear regression model in which age and pegivirus co-infection status are the independent variables and Ct is the dependent variable. **G.** Receiver operating characteristic (ROC) curve for the multivariate logistic regression model in which age, pegivirus co-infection status, Ct, and ribavirin treatment are the independent variables and LF outcome is the dependent variable. 0.84, area under the curve. **HI.** Graphical summary of the average causal mediation effect (ACME), average direct effect (ADE), and total effect of age (**H**; $p = 2 \times 10^{-16}$) and pegivirus C co-infection status (**I**; $p = 0.02$) on LF outcome, where Ct is the mediator variable. Adjusted p-values and confidence intervals were determined using bootstrapping. ***, $p < 0.001$. *, $p < 0.05$. **J.** Number of reads mapped to Pegivirus C vs. Lassa virus. Correlation coefficient = -0.02, $p = 0.63$ via permutation.

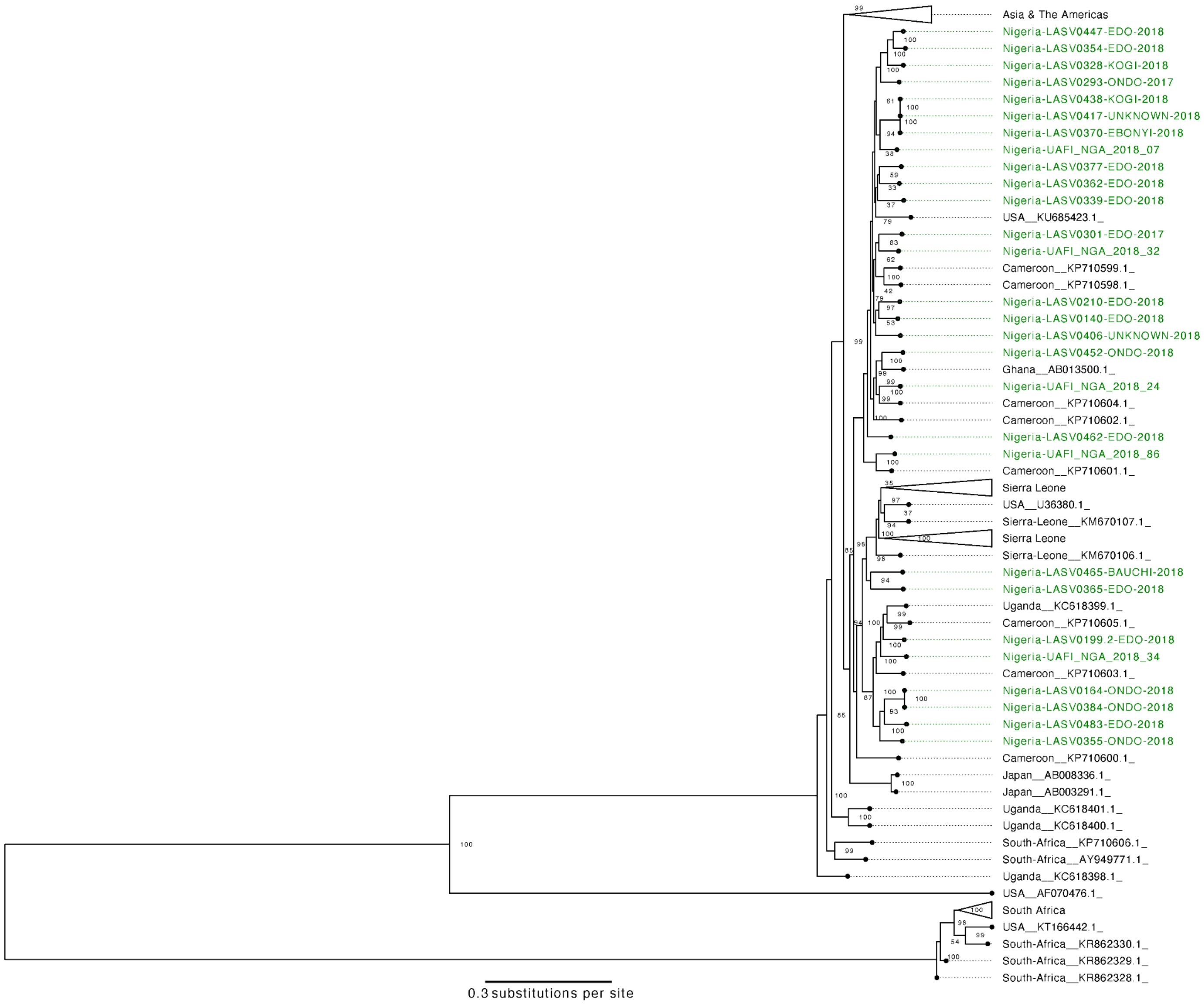

Supplementary Figure 5. Pegivirus C genetic diversity. Maximum likelihood phylogenetic tree with 28 new genomes (green) alongside 130 full-length, annotated sequences. Generated from whole-genome alignment (9,942 bp). Bootstrap values of key nodes are shown.

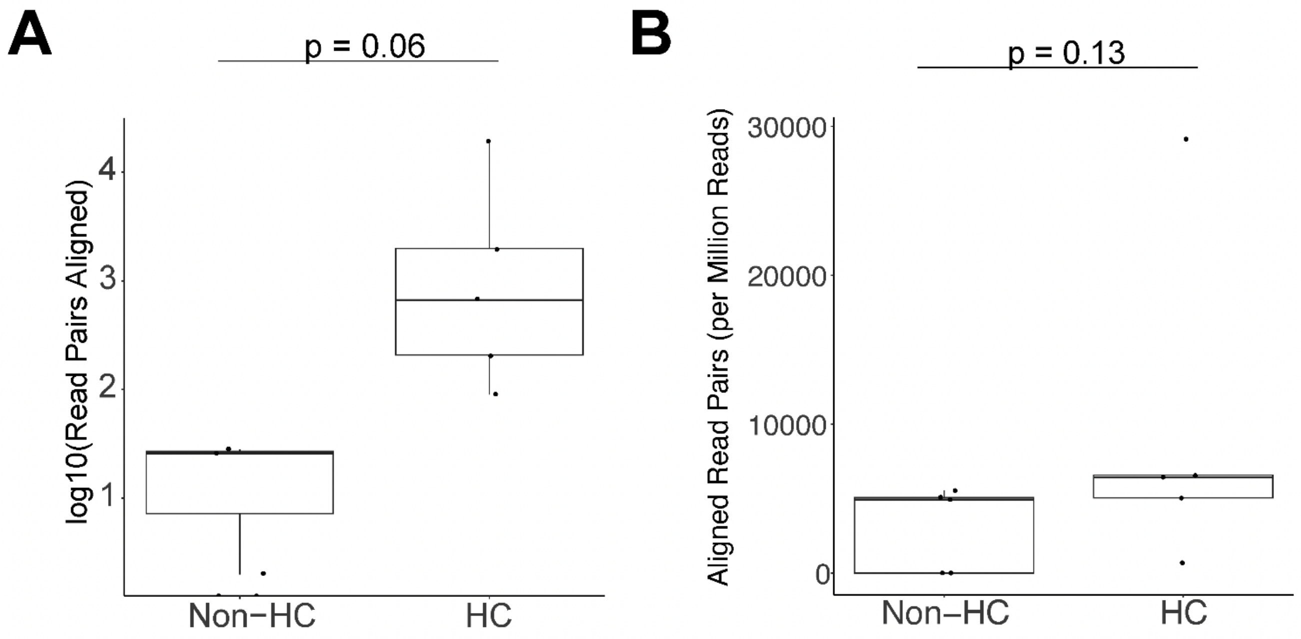

Supplementary Figure 6. Monkeypox virus (MPXV) sequencing via unbiased metagenomics and hybrid capture (HC). AB. Read pairs aligned to the MPXV genome (**A**) and read pairs per million reads aligned to the MPXV genome (**B**) with unbiased metagenomics (Non-HC) and with pan-viral enrichment probes, enabling HC. P-values via two-sided Wilcoxon signed rank test (N = 5 samples). Boxplots display the first, second, and third quartiles, with whiskers extending to the data point that is maximally 1.5 times the interquartile range from the first (lower whisker) or third (upper whisker) quartile.

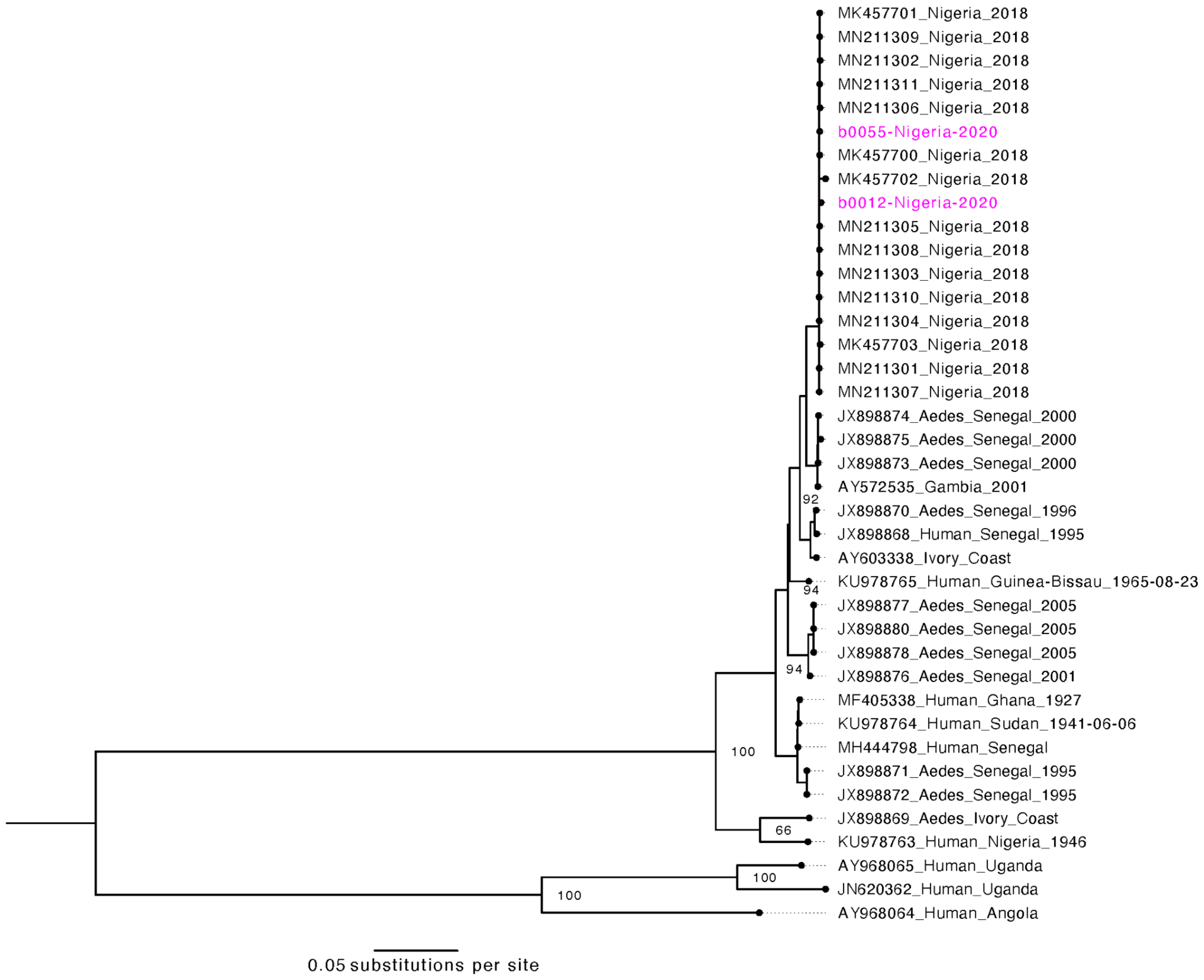

Supplementary Figure 7: Yellow fever virus (YFV) genetic diversity. Maximum likelihood phylogenetic tree with 2 new sequences (pink) alongside contextual African sequences. Generated from whole-genome alignment (10,877 bp). Bootstrap values for key nodes are shown.

Supplementary Tables

Supplementary Table 1: Sequencing batches. Sequencing was conducted at two sites (Nigeria, using the MiSeq; Massachusetts, using the other machines) over three years. Batch-specific controls are listed. CDI, combinatorial dual indexes. ERCCs, External RNA Controls Consortium (synthetic sequence spike-ins). LASV, Lassa virus. MPXV, monkeypox virus. UDI, unique dual indexes. YFV, yellow fever virus.

Samples	Batch ID	No. Samples	Machine	ERCCs	Indices	Date	Water Control	K5562 Control	Positive Control	Positive Ctrl Virus
LASV-	121935	99	Novaseq	yes	UDI	10/25/18	aWATER_1, aWATER_2, aWATER_3, aWATER_4, aWATER_5	aK562_1, aK562_2, aK562_3	aVIRUS_1, aVIRUS_2, aVIRUS_3	Ebola (Makona)
LASV+	180406	7	HiSeq2500	yes	CDI	4/6/18	dWATER_05_180406	dK562_02_180406		
LASV+	180419	145	HiSeq2500	yes	CDI	4/19/18	dWATER_01_180419, dWATER_02_180419, dWATER_03_180419, dWATER_04_180419, dWATER_05_180419, dWATER_06_180419, dWATER_07_180419, dWATER_08_180419, dWATER_09_180419	dK562_01_180419, dK562_02_180419, dK562_03_180419, dK562_04_180419	dMAK_01_180419, dMAK_02_180419, dMAK_03_180419	Ebola (Makona)
LASV+	180601	108	HiSeq2500	yes	CDI	6/1/18	dWATER_10_180601, dWATER_11_180601, dWATER_12_180601, dWATER_13_180601, dWATER_14_180601, dWATER_15_180601	dK562_05_180601, dK562_06_180601, dK562_07_180601	dMAK_05_180601, dMAK_06_180601, dMAK_07_180601	Ebola (Makona)
LASV+	180627	149	HiSeq2500	yes	CDI	6/27/18	dWATER_16_180627, dWATER_17_1	dK562_08_180627,	dMAK_08_180627, dMAK_09_1	Ebola (Makona)

							80627, dWATER_18_180627, dWATER_19_180627, dWATER_20_180627, dWATER_21_180627, dWATER_22_180627, dWATER_23_180627	dK562_09, dK562_10_180627, dK562_11_180627	80627, dMAK_10_180627, dMAK_11_180627	
LASV+	180315	2	MiSeq	no	CDI	3/15/18	cn053		180315_mumps	Mumps
MPXV+	180502	5	MiSeq	no	CDI	5/2/18				
LASV+	180802_M03472	1	MiSeq	no	CDI	8/2/18	cn075	ck080, ck232	cm074, cm078, cm082, cm231	Ebola (Makona)
LASV+	180802_M50197	3	MiSeq	no	CDI	8/2/18	cn083, cn095	ck092		
LASV+	180809	1	MiSeq	no	CDI	8/9/18	cn103, cn105	ck096, ck099	cm098	Ebola (Makona)
LASV+	180817	1	MiSeq	no	CDI	8/17/18		180817_M03472_K562	cm120	Ebola (Makona)
LASV+	181018	1	MiSeq	no	CDI	10/18/18	cn150			
unknown	190321	1	MiSeq	no	CDI	3/21/19	cn216	ck217		
LASV+	190510	5	MiSeq	no	CDI	5/10/19	cn224	ck223		
LASV+	190524	12	MiSeq	no	CDI	5/24/19	c0172	c0168		
LASV+	190703	11	MiSeq	no	CDI	7/3/19		c0185		
LASV+; unknown	190708	3	MiSeq	no	CDI	7/8/19	cn193	ck192		
LASV+	190710	2	MiSeq	no	CDI	7/10/19	cn199	ck198		
LASV+	191024	1	MiSeq	no	CDI	10/24/19	191024_M50197_NEG	191024_M50197_K562		
LASV+	191213	3	MiSeq	no	CDI	12/13/19	cn204	ck205		
unknown	200121	3	MiSeq	no	CDI	1/21/20	200121_M50197_NEG	200121_M50197_K562		
LASV+; YFV+	200213	6	MiSeq	no	CDI	2/13/20	cn214	ck215		

Benue unknown	200220	12	MiSeq	no	CDI	2/20/20	bw040	bk041		
LASV+	200303	2	MiSeq	no	CDI	3/3/20		ck229		
unknown	200314	3	MiSeq	no	CDI	3/14/20	bn047	bk048	bp049	Lassa
YFV+	201123_M03472	7	MiSeq	no	CDI	11/23/20	201123_M03472_NE	201123_M03472_K13		

Supplementary Table 2: Positive controls. Positive controls displayed evidence of the spiked-in virus in 20 of 21 cases.

Positive Control	Batch	Viral Spike-in	Pathogen Assigned	Reads Assigned	Total Reads
aVIRUS_1	121935	Ebola (Makona)	Zaire_ebolavirus	547781	19466424
aVIRUS_2	121935	Ebola (Makona)	Zaire_ebolavirus	526919	12517310
aVIRUS_3	121935	Ebola (Makona)	Zaire_ebolavirus	1037052	22901606
dMAK_01_180419	180419	Ebola (Makona)	Zaire_ebolavirus	1002818	19062322
dMAK_02_180419	180419	Ebola (Makona)	Zaire_ebolavirus	1928268	66064684
dMAK_03_180419	180419	Ebola (Makona)	Zaire_ebolavirus	891347	34899456
dMAK_05_180601	180601	Ebola (Makona)	Zaire_ebolavirus	428714	8873320
dMAK_06_180601	180601	Ebola (Makona)	Zaire_ebolavirus	0	10527226
dMAK_07_180601	180601	Ebola (Makona)	Zaire_ebolavirus	313739	6559492
dMAK_08_180627	180627	Ebola (Makona)	Zaire_ebolavirus	2683431	25143424
dMAK_09_180627	180627	Ebola (Makona)	Zaire_ebolavirus	1758026	22426226
dMAK_10_180627	180627	Ebola (Makona)	Zaire_ebolavirus	903252	7835996
dMAK_11_180627	180627	Ebola (Makona)	Zaire_ebolavirus	478253	6593430
180315_mumps	180315	Mumps	Mumps_orthorubulavirus	244	622874
cm074	180802_M03472	Ebola (Makona)	Zaire_ebolavirus	95253	681398
cm078	180802_M03472	Ebola (Makona)	Zaire_ebolavirus	178774	1621416
cm082	180802_M03472	Ebola (Makona)	Zaire_ebolavirus	285686	3057596
cm231	180802_M03472	Ebola (Makona)	Zaire_ebolavirus	188989	1472294
cm098	180809	Ebola (Makona)	Zaire_ebolavirus	216695	1602638
cm120	180817	Ebola (Makona)	Zaire_ebolavirus	282073	3025580
bp049	200314	Lassa	Lassa_mammarenavirus	18065	2506984

Supplementary Table 3: Causal mediation analyses. Nonparametric bootstrap 95% confidence intervals (95% CI) were derived to estimate the average causal mediation effect (ACME), average direct effect (ADE), and total effect of age and of pegivirus C co-infection status on Lassa Fever survival, where Ct is the mediator variable. Adjusted p-values via bootstrapping. ***, $p < 0.001$. *, $p < 0.05$.

**Independent Variable**	**Estimate**	**95% CI**	**P-value**
**Age**			
Total Effect	0.001	(-0.001) - 0.00	0.17
Average Causal Mediation Effect	0.001	0.0004 - 0.00	2×10^{-16}***
Average Direct Effect	0.00006	(-0.002) - 0.00	1.00
Proportion Mediated	0.95	(-5.23) - 8.35	0.17
**Pegivirus**			
Total Effect	-0.07	(-0.17) - 0.07	0.27
Average Causal Mediation Effect	-0.05	(-0.09) - (-0.01)	0.02*
Average Direct Effect	-0.03	(-0.15) - 0.13	0.70
Proportion. Mediated	0.62	(-6.12) - 8.63	0.29

Supplementary Table 4: Human immunodeficiency virus 1 (HIV-1) subtypes. LASV, Lassa virus.

Sample Name	Subtype Assignment	LASV Status
UAFI_NGA_2018_37	CRF02_AG	Negative
UAFI_NGA_2018_39	B	Negative
UAFI_NGA_2018_68	undetermined	Negative
LASV0247-EDO-2017	C	Positive
LASV0332-ONDO-2018	G	Positive
LASV0335-ONDO-2018	G	Positive
LASV0479-ONDO-2018	CRF02_AG	Positive

Supplementary Table 5. Common pathogens panel qPCR primers. YFV = yellow fever virus, WNV = West Nile virus, ZIKV = Zika virus, CHIKV = Chikungunya virus, ONNV = O'nyong-nyong virus, LASV = Lassa virus, EBOV = Ebola virus.

**Pathogen**	**Forward and Reverse Primers**
Pan-Flavivirus	TACAACATGATGGGAAAGAGAGAGAARAA<br>GTGTCCCAKCCRGCTGTGTCATC
Pan-Alphavirus	YAGAGCDTTTTCGCAYSTRGCHW<br>CATRAANKGNGTNGTRTCRAANCCDAYCC
YFV	GCTAATTGAGGTGYATTGGTCTGC<br>CTGCTAATCGCTCAAMGAACG
WNV	GGGCCTTCTGGTCGTGTTC<br>GATCTTGGCYGTCCACCTC
ZIKV	AARTACACATACCARAACAAAGTG GT<br>TCCRCTCCCYCTYTGGTCTTG
CHIKV	GACAATGCGCGCGGTACC<br>TGTTGTTTTGTGGCGCCT
ONNV	CAGTGATCCCGAACACGGTG<br>CCACATAAATGGGTAGACGCC
Pan-Dengue	TTGAGTAAACYRTGCTGCCTGTAGCTC<br>GAGACAGCAGGATCTCTGGTCTYTC
LASV	YACAGGGTCYTCTGGWCGACC<br>RATGATGCARCTTGACCCAAG<br><br>Altona Diagnostics RealStar® Lassa Virus RT-PCR Kit 2.0
EBOV	GTCGTTCCAACAATCGAGCG<br>CGTCCCGTAGCTTTRGCCAT

Supplementary Table 6. Monkeypox virus (MPXV) samples. 71 samples with suspicion for MPXV were received, and were tested for MPXV using qPCR. Ct values are provided. 5 samples with evidence of PCR positivity were sequenced. *B6R* primers were developed by Li et al.[2]

Identifier	Cycle Threshold	Viral Titer (copies/uL)	Sequencing Identifier	MPXV Reads	Assembled Genome Length	Genome Percent Assembly
MPXV001	-	-	-	-	-	-
MPXV002	-	-	-	-	-	-
MPXV003	32.76	151.52	b0024	210	5798	0.029
MPXV004	-	-	-	-	-	-
MPXV005	36.80	10.25	-	-	-	-
MPXV006	-	-	-	-	-	-
MPXV007	-	-	-	-	-	-
MPXV008	35.17	30.70	-	-	-	-
MPXV009	38.36	3.64	-	-	-	-
MPXV010	-	-	-	-	-	-
MPXV011	-	-	-	-	-	-
MPXV012	-	-	-	-	-	-
MPXV013	-	-	-	-	-	-
MPXV014	35.85	20.32	-	-	-	-
MPXV015	-	-	-	-	-	-
MPXV016	-	-	-	-	-	-
MPXV017	-	-	-	-	-	-
MPXV018	-	-	-	-	-	-
MPXV019	-	-	-	-	-	-
MPXV020	36.79	1.84	-	-	-	-
MPXV021	34.47	-	-	-	-	-
MPXV022	38.64	1.45	-	-	-	-
MPXV023	-	-	-	-	-	-
MPXV024	-	-	-	-	-	-
MPXV025	34.81	-	-	-	-	-
MPXV026	-	-	-	-	-	-
MPXV027	-		-	-	-	-
MPXV028	29.69	76.26	-	-	-	-
MPXV029	28.05	188.70	-	-	-	-
MPXV030	32.48	13.42	-	-	-	-
MPXV031	31.12	28.65	-	-	-	-

MPXV032	26.06	856.10	b0025	692	13817	0.070
MPXV033	34.90	0.47	-	-	-	-
MPXV034	35.75	-	-	-	-	-
MPXV035	34.41	1.00	-	-	-	-
MPXV036	41.33	1.05	-	-	-	-
MPXV037	39.05	-	-	-	-	-
MPXV038	-	-	-	-	-	-
MPXV039	-	-	-	-	-	-
MPXV040	-		-	-	-	-
MPXV041	-	-	-	-	-	-
MPXV042	35.90	0.0038	-	-	-	-
MPXV043	31.14	23.90	-	-	-	-
MPXV044	37.21	0.21	-	-	-	-
MPXV045	-		-	-	-	-
MPXV046	41.63	-	-	-	-	-
MPXV047	-	-	-	-	-	-
MPXV048	-	-	-	-	-	-
MPXV049	35.76	-	-	-	-	-
MPXV050	-	-	-	-	-	-
MPXV051	-	-	-	-	-	-
MPXV052	29.92	49.96	b0026	90	2813	0.014
MPXV053	-	-	-	-	-	-
MPXV054	35.31	2.38	-	-	-	-
MPXV055	29.63	501.80	-	-	-	-
MPXV056	26.59	663.80	b0027	1988	62552	0.317
MPXV057	-	-	-	-	-	-
MPXV058	-	-	-	-	-	-
MPXV059	-	-	-	-	-	-
MPXV060	39.41	-	-	-	-	-
MPXV061	37.00	15.96	-	-	-	-
MPXV062	31.93	0.95	-	-	-	-
MPXV063	-	-	-	-	-	-
MPXV064	35.60	-	-	-	-	-
MPXV065	-	-	-	-	-	-
MPXV066	34.79	0.58	-	-	-	-
MPXV067	28.99	106.40	b0028	19776	37573	0.190

MPXV068	-	-	-	-	-	-
MPXV069	-	-	-	-	-	-
MPXV070	37.57	-	-	-	-	-
MPXV071	-	-	-	-	-	-

Supplementary Table 7. Metadata associated with 8 samples sequenced from patients with unusual clinical presentations. IV, intravenous. NA, not applicable (i.e., missing information).

Sample Name	Date of Sample Isolation	Age	Sex	Case Information
b0003	Jul-Sept, 2019	NA	NA	NA
b0009	Oct-Dec, 2019	1-5	Male	• 2 weeks of weakness in upper and lower limb<br>• Left upper and lower limb strength = 1<br>• Generalized significant lymphadenopathy and hepatosplenomegaly<br>• Ct scan showed evidence of right hemisphere stroke
b0010	Oct-Dec, 2019	6-10	Female	• 1 week of headache, joint pain, and fever followed by 8 days of unconsciousness<br>• Received IV antibiotics<br>• Sample sent 11 days after admission<br>• Clinical suspicion for viral meningoencephalitis
b0011	Oct-Dec, 2019	40-45	Male	• Jaundice
b0013	Jan-Mar, 2020	1-5	Male	• Fever<br>• Convulsions
b0044	Jan-Mar, 2020	NA	NA	NA
b0045	Jan-Mar, 2020	NA	NA	NA
b0046	Jan-Mar, 2020	NA	NA	NA

Supplementary References

1. Nikisins, S. *et al.* International external quality assessment study for molecular detection of Lassa virus. *PLoS Negl. Trop. Dis.* **9**, e0003793 (2015).
2. Li, Y., Olson, V. A., Laue, T., Laker, M. T. & Damon, I. K. Detection of monkeypox virus with real-time PCR assays. *J. Clin. Virol.* **36**, 194–203 (2006).
