# Supplementary material for: Metagenomic surveillance uncovers diverse and novel viral taxa in febrile patients from Nigeria
Source: Nat Commun. 2023 Aug 4;14:4693. doi: 10.1038/s41467-023-40247-4 (PMC10403498; doi:10.1038/s41467-023-40247-4)
Supplement: Supplementary file 3 — Description of Additional Supplementary Files [file 41467_2023_40247_MOESM3_ESM.pdf]

Description of Additional Supplementary Files

Supplementary Data 1: Sample metadata, RT-qPCR data, and sequencing data for all samples. The file contains (i) sample information, including collection date, state, and NCBI BioSample accession codes; (ii) taxonomic classification of viral reads by Microsoft Premonition; (iii) viral genome assembly data; (iv) viruses detected in each sample; (v) reference sequences used for reference-guided genome assembly; (vi) RT-qPCR data for all samples screened with the common pathogens panel.
