# Supplementary material for: Metagenomic surveillance uncovers diverse and novel viral taxa in febrile patients from Nigeria
Source: Nat Commun. 2023 Aug 4;14:4693. doi: 10.1038/s41467-023-40247-4 (PMC10403498; doi:10.1038/s41467-023-40247-4)
Supplement: Supplementary file 5 — Reporting Summary [file 41467_2023_40247_MOESM5_ESM.pdf]

nature portfolio

Corresponding author(s): Christian Happi, Pardis Sabeti, Katherine Siddle

Last updated by author(s): Jun 11, 2023

Reporting Summary

Nature Portfolio wishes to improve the reproducibility of the work that we publish. This form provides structure for consistency and transparency in reporting. For further information on Nature Portfolio policies, see our Editorial Policies and the Editorial Policy Checklist.

Statistics

For all statistical analyses, confirm that the following items are present in the figure legend, table legend, main text, or Methods section.

n/a	Confirmed	
☐	☒	The exact sample size (*n*) for each experimental group/condition, given as a discrete number and unit of measurement
☐	☒	A statement on whether measurements were taken from distinct samples or whether the same sample was measured repeatedly
☐	☒	The statistical test(s) used AND whether they are one- or two-sided<br>*Only common tests should be described solely by name; describe more complex techniques in the Methods section.*
☐	☒	A description of all covariates tested
☐	☒	A description of any assumptions or corrections, such as tests of normality and adjustment for multiple comparisons
☐	☒	A full description of the statistical parameters including central tendency (e.g. means) or other basic estimates (e.g. regression coefficient) AND variation (e.g. standard deviation) or associated estimates of uncertainty (e.g. confidence intervals)
☐	☒	For null hypothesis testing, the test statistic (e.g. *F*, *t*, *r*) with confidence intervals, effect sizes, degrees of freedom and *P* value noted<br>*Give P values as exact values whenever suitable.*
☒	☐	For Bayesian analysis, information on the choice of priors and Markov chain Monte Carlo settings
☒	☐	For hierarchical and complex designs, identification of the appropriate level for tests and full reporting of outcomes
☐	☒	Estimates of effect sizes (e.g. Cohen's *d*, Pearson's *r*), indicating how they were calculated

Our web collection on statistics for biologists contains articles on many of the points above.

Software and code

Policy information about availability of computer code

Data collection	No software was used for data collection.
Data analysis	Open source software used in this study is available at https://github.com/broadinstitute/viral-ngs (i.e., pipelines for viral genomic analyses; v2.1.8) and at doi:10.5281/zenodo.8020941 (i.e., code for statistical analyses; developed in R v4.1.1 with packages bda v15.2.5, mediation v4.5.0, ROCR v1.0-11, stats v4.1.1, and tidyverse v2.0.0). Information about the Microsoft Premonition metagenomics pipeline is available at https://microsoft.com/premonition. Individuals can access the pipeline ahead of its public release by clicking the “Contact us for availability” button and mentioning this work, or by emailing Simon Frost at Frost.Simon@microsoft.com.<br><br>Viral genotyping tools include the following: Hepatitis A (https://www.rivm.nl/mpf/typingtool/hav/), Enterovirus B (https://www.rivm.nl/mpf/typingtool/enterovirus/), HIV (Stanford University HIV Drug Resistance Database; https://hivdb.stanford.edu/hivdb/), and Hepatitis B (https://www.genomedetective.com/app/typingtool/hbv/).<br><br>Finally, phylogenetics was conducted using MAFFT, IQ-TREE v2.0.3 (Lassa virus) or v1.6.12 (all other viruses), Geneious Prime v2023.0.4 (www.geneious.com), and FigTree v1.4.4.

For manuscripts utilizing custom algorithms or software that are central to the research but not yet described in published literature, software must be made available to editors and reviewers. We strongly encourage code deposition in a community repository (e.g. GitHub). See the Nature Portfolio guidelines for submitting code & software for further information.

Data

Policy information about availability of data

All manuscripts must include a data availability statement. This statement should provide the following information, where applicable:

- Accession codes, unique identifiers, or web links for publicly available datasets
- A description of any restrictions on data availability
- For clinical datasets or third party data, please ensure that the statement adheres to our policy

The raw reads and complete pathogen genomes generated in this study have been deposited in the Sequence Read Archive (SRA) and NCBI GenBank, respectively, under BioProject accession codes PRJNA824010 (https://www.ncbi.nlm.nih.gov/bioproject/?term=PRJNA824010) and PRJNA436552 (https://www.ncbi.nlm.nih.gov/bioproject/?term=PRJNA436552). Sample metadata (collection date, state, age, sequencing machine, sequencing batch, etc.), metagenomic read classification data for all samples and controls, viral genome assembly data, reference sequence accession numbers, and RT-qPCR results generated in this study are provided in the Supplementary Data file.

Research involving human participants, their data, or biological material

Policy information about studies with human participants or human data. See also policy information about sex, gender (identity/presentation), and sexual orientation and race, ethnicity and racism.

Reporting on sex and gender	We provide participant self-reported sex.
Reporting on race, ethnicity, or other socially relevant groupings	We do not report participant race or ethnicity. The study was conducted in Nigeria, where hundreds of distinct ethnic groups exist.
Population characteristics	Among the cohort with Lassa Fever (LF), we collected age, sex, and pregnancy status. The median (IQR) age was 31 (22-45). 166/399 (41.9%) of individuals were female. 4/95 (4.2%) of females with known pregnancy status were pregnant. We do not have cohort demographics for the three outbreaks. We provide age and sex for the 8 unusual clinical cases in the supplementary tables. Because the 8 cases present with a range of symptoms and etiologies, we do not calculate summary statistics.
Recruitment	Samples were collected from participants who sought medical care and were febrile; therefore, our findings are applicable to individuals with these characteristics.<br><br>For individuals to be considered for Lassa Fever, we required: (a) fever ≥38 °C and no improvement after 2 days of antimalarials or antibiotics, or (b) fever ≥38 °C with at least one LF-associated symptom: bleeding from mucosal surfaces or injection sites, deafness, conjunctivitis, facial edema, hypotension, spontaneous abortion, seizures, encephalopathy, or acute kidney injury. It is possible that this case definition would miss asymptomatic or mild cases of Lassa Fever, though such individuals are less likely to seek medical attention.
Ethics oversight	Institutional review boards of Irrua Specialist Teaching Hospital (ISTH; Irrua, Nigeria), Redeemer's University, and Harvard University (Cambridge, Massachusetts) assessed and approved the study before samples suspected to contain Lassa virus were collected at ISTH. De-identified clinical samples and demographic and clinical data were collected under (i) a waiver of consent, approved by the ISTH Research Ethics Committee, or (ii) under the written informed consent of participants who signed up to participate in a separate study that analyzed human genetic material. The waiver of consent enables the analysis of pathogen genomic data and de-identified demographic and clinical data, but not the analysis of human genetic material. For the purposes of the work in this manuscript, the sample sets are equivalent in terms of data availability.<br><br>For the outbreaks, the Nigerian Centre for Disease Control collected samples. As a regulatory body for public health, the NCDC can both collect samples and send them to the African Centre of Excellence for Genomics of Infectious Diseases (ACEGID) at Redeemer's University for sequencing in the context of public health emergencies. Therefore, these samples were collected under disease surveillance conditions from a recognized public health authority, rather than in a research-driven context.<br><br>For the unusual clinical presentations, samples from Federal Teaching Hospital Abakaliki (FETHA) and Federal Medical Center (FMC) Owo were received via a study approved by the Institutional Review Board (IRB) at the National Health Research Ethics Committee (Nigeria).

Note that full information on the approval of the study protocol must also be provided in the manuscript.

Field-specific reporting

Please select the one below that is the best fit for your research. If you are not sure, read the appropriate sections before making your selection.

☒ Life sciences ☐ Behavioural & social sciences ☐ Ecological, evolutionary & environmental sciences

For a reference copy of the document with all sections, see nature.com/documents/nr-reporting-summary-flat.pdf

Life sciences study design

All studies must disclose on these points even when the disclosure is negative.

Sample size	No sample size calculations were performed. Sample size is a function of clinical case counts and sample availability.
Data exclusions	We removed samples that did not successfully sequence (i.e., those with < 1000 total reads).
Replication	We successfully reproduced the cases of diagnostic failure in a supplemental figure. Some samples were sequenced in duplicate, and the sample preparation that yielded more total reads was retained.
Randomization	Randomization is not relevant to this study as it is observational, and aims to use metagenomics in real-world settings to characterize etiologies of disease, identify risk factors for death following Lassa virus infection, discover novel viral taxa, & generate public health insights via the study of pathogen transmission.
Blinding	Blinding is not relevant to this study as it is observational (i.e., no interventions were conducted).

Reporting for specific materials, systems and methods

We require information from authors about some types of materials, experimental systems and methods used in many studies. Here, indicate whether each material, system or method listed is relevant to your study. If you are not sure if a list item applies to your research, read the appropriate section before selecting a response.

Materials & experimental systems

n/a	Involved in the study	
☒	☐	Antibodies
☒	☐	Eukaryotic cell lines
☒	☐	Palaeontology and archaeology
☒	☐	Animals and other organisms
☒	☐	Clinical data
☒	☐	Dual use research of concern
☒	☐	Plants

Methods

n/a	Involved in the study	
☒	☐	ChIP-seq
☒	☐	Flow cytometry
☒	☐	MRI-based neuroimaging
